# Supplementary material for: SARS-CoV-2 Omicron BA.1 and BA.2 are attenuated in rhesus macaques as compared to Delta
Source: Sci Adv. 2022 Nov 18;8(46):eade1860. doi: 10.1126/sciadv.ade1860 (PMC9674298; doi:10.1126/sciadv.ade1860)
Supplement: Supplementary file 1 — Table S1 Figs. S1 to S6 [file sciadv.ade1860_sm.pdf]

Science Advances
AAAS

Supplementary Materials for

SARS-CoV-2 Omicron BA.1 and BA.2 are attenuated in rhesus macaques as compared to Delta

Neeltje van Doremalen *et al.*

Corresponding author: Neeltje van Doremalen, neeltje.vandoremalen@nih.gov;
Kyle Rosenke, kyle.rosenke@nih.gov

Sci. Adv. **8**, eade1860 (2022)
DOI: 10.1126/sciadv.ade1860

This PDF file includes:

Table S1
Figs. S1 to S6

Table S1. NHPs were inoculated with SARS-CoV-2 VOCs Delta AY.106 (hCoV-19/USA/MD-HP05647/2021, EPI_ISL_2331496), Omicron BA.1 (hCoV-19/USA/GA-EHC-2811C/2021, EPI_ISL_7171744), or Omicron BA.2 (hCoV-19/Japan/UT-NCD1288-2N/2022, EPI_ISL_9595604). No substitutions in the S protein compared to published sequence were found. All amino acid substitutions compared to ancestral S protein Wuhan are detailed below.

AA	Wuhan	Delta AY.106	Omicron BA.1	Omicron BA.2	Region	
19	T	R	T	I	S1	NTD
24	L	L	L	-		
25	P	P	P	-		
26	P	P	P	-		
27	A	A	A	S		
67	A	A	V	A		
69	H	H	-	H		
70	V	V	-	V		
95	T	I	I	T		
142	G	D	D	D		
143	V	V	-	V		
144	Y	Y	-	Y		
145	Y	Y	-	Y		
156	E	-	E	E		
157	F	-	F	F		
158	R	G	R	R		
211	N	N	-	N		
212	L	L	I	L		
213	V	V	V	G		
214	R	R	REPE	R		
255	S	F	S	S		
339	G	G	D	D		RBD
371	S	S	L	F		
373	S	S	P	P		
375	S	S	F	F		

376	T	T	T	A		
405	D	D	D	N		
408	R	R	R	S		
417	K	K	N	N		
440	N	N	K	K		
446	G	G	S	G		
452	L	R	L	L		
477	S	S	N	N		
478	T	K	K	K		
484	E	E	A	A		
493	Q	Q	R	R		
496	G	G	S	G		
498	Q	Q	R	R		
501	N	N	Y	Y		
505	Y	Y	H	H		
547	T	T	K	T		
614	D	G	G	G		
655	H	H	Y	Y		
679	N	N	K	K		
681	P	R	H	H		
764	N	N	K	K		
796	D	D	Y	Y		Fusion
856	N	N	K	N		
950	D	N	D	D	S2	
954	Q	Q	H	H		HR1
969	N	N	K	K		
981	L	L	F	L		

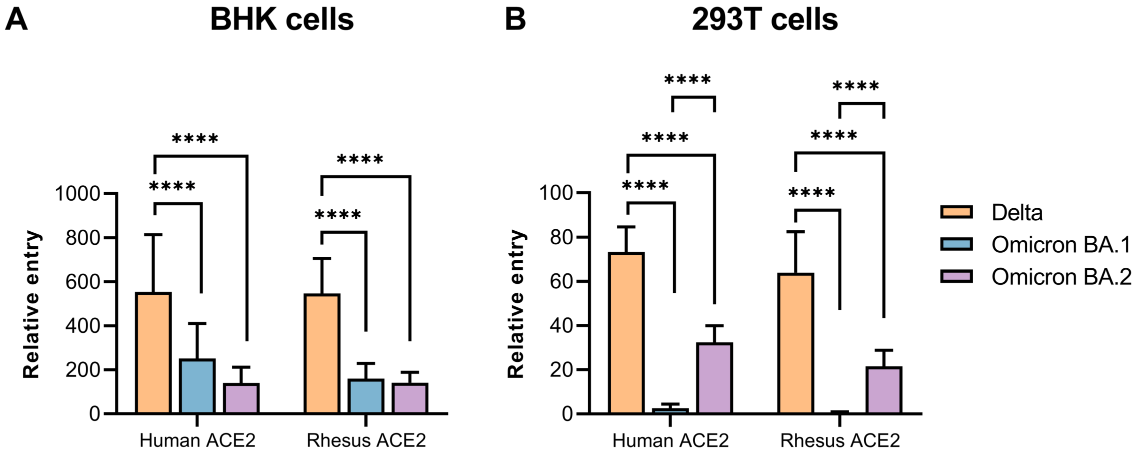

Figure S1. Comparison of entry of SARS-CoV-2 S proteins to human and rhesus macaque ACE2. BHK cells were transfected with either human ACE2 or rhesus ACE2 and subsequently infected with pseudotyped VSV reporter particles with the S proteins of Delta, Omicron BA.1, or Omicron BA.2. Luciferase expression was measured, and relative entry of the VOCs was calculated over no spike pseudotype. N=16, combined from two separate experiments. Statistical analysis was performed using a one-way ANOVA with Tukey's multiple comparisons test.

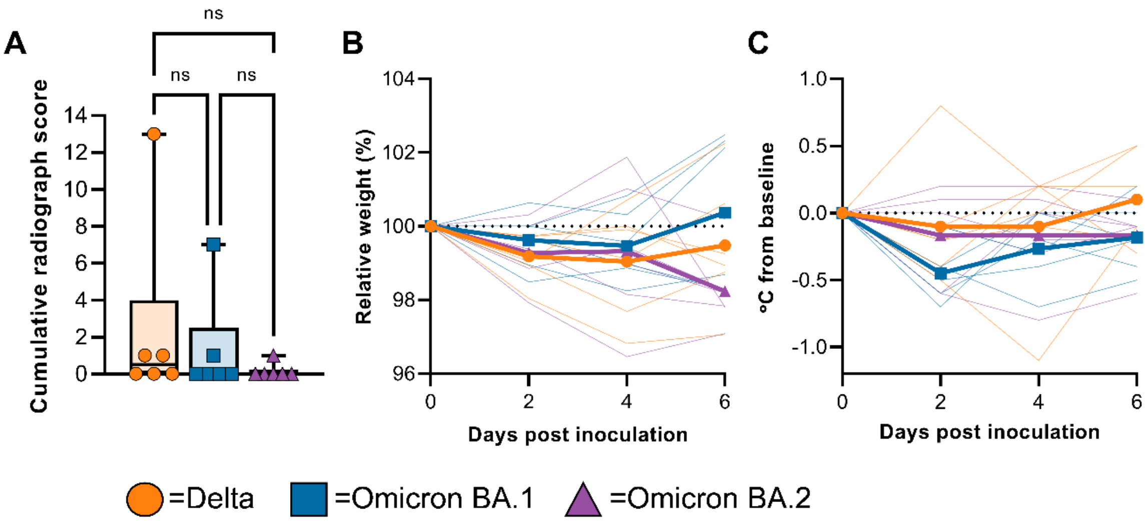

Figure S2. Limited signs of disease on radiographs, weight and temperature. (A) Minimum-to-maximum boxplot of ventrodorsal radiographs taken on exam days. Individual lobes were scored by a clinical veterinarian according to a standardized scoring system and totaled. Statistical analysis was performed using a Kruskal-Wallis test with Dunn's multiple comparisons. (B) The relative weight compared to the day of challenge (dotted line) is shown per group (median, thick line) as well as per individual (thin lines). (C) Body temperature is indicated as deviation from baseline at the day of challenge (dotted line) and shown per group (median, thick line) as well as per individual (thin lines).

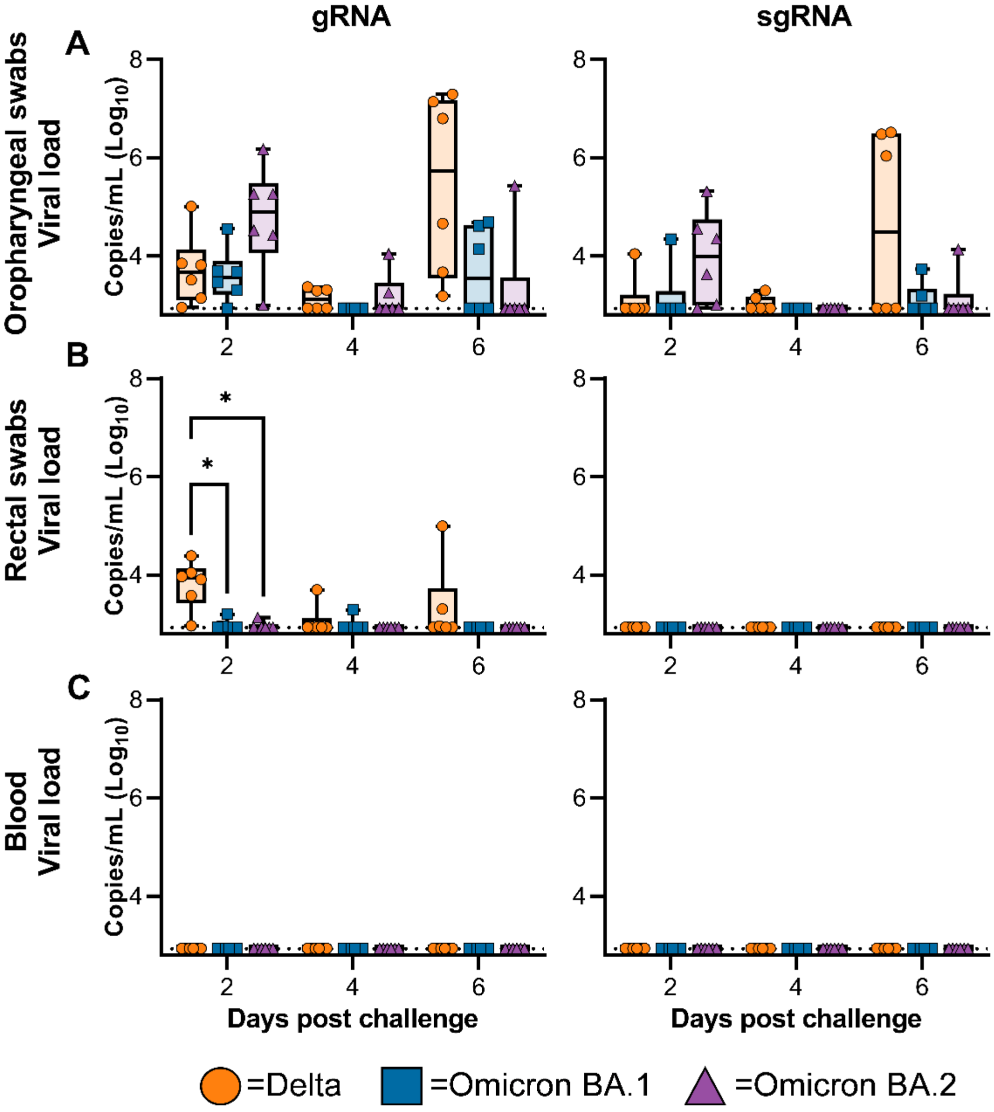

Figure S3. Limited differences in shedding of viral RNA were detected in throat swabs, rectal swabs, and blood. Boxplot (minimum-to-maximum) of viral gRNA (left panel) and sgRNA (right panel) in throat swabs (A), rectal swabs (B), and blood (C) taken on 2-, 4-, and 6-dpi. Statistical significance was determined via a two-way ANOVA with the Geisser-Greenhouse correction followed by the Tukey test for multiple comparisons.

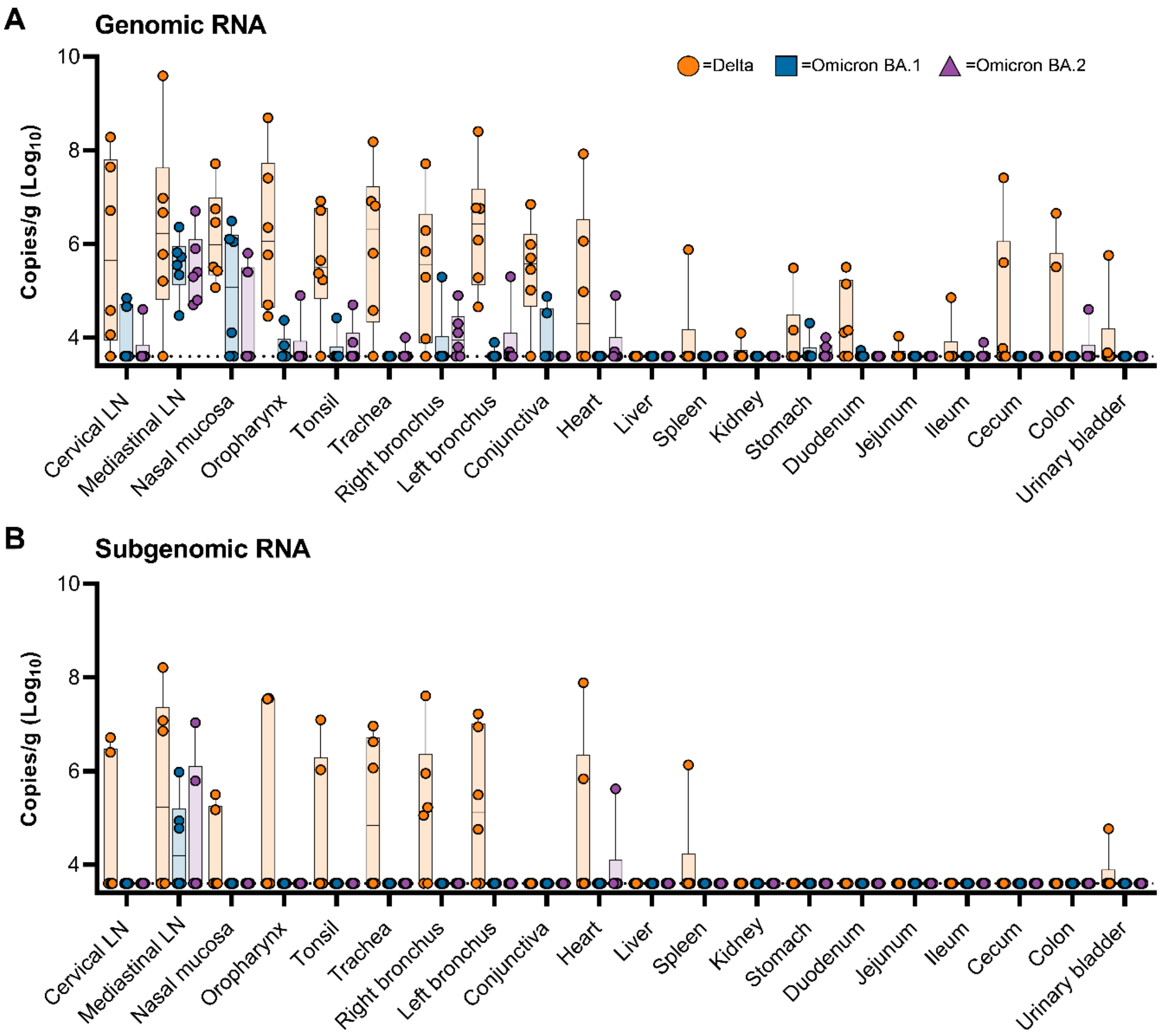

Figure S4. Viral loads in non-respiratory tissues is limited for Omicron BA.1 and BA.2 inoculated animals on 6-dpi. (A) Boxplot (minimum-to-maximum) of gRNA detected in tissues. (B) Boxplot (minimum-to-maximum) of sgRNA detected in tissues.

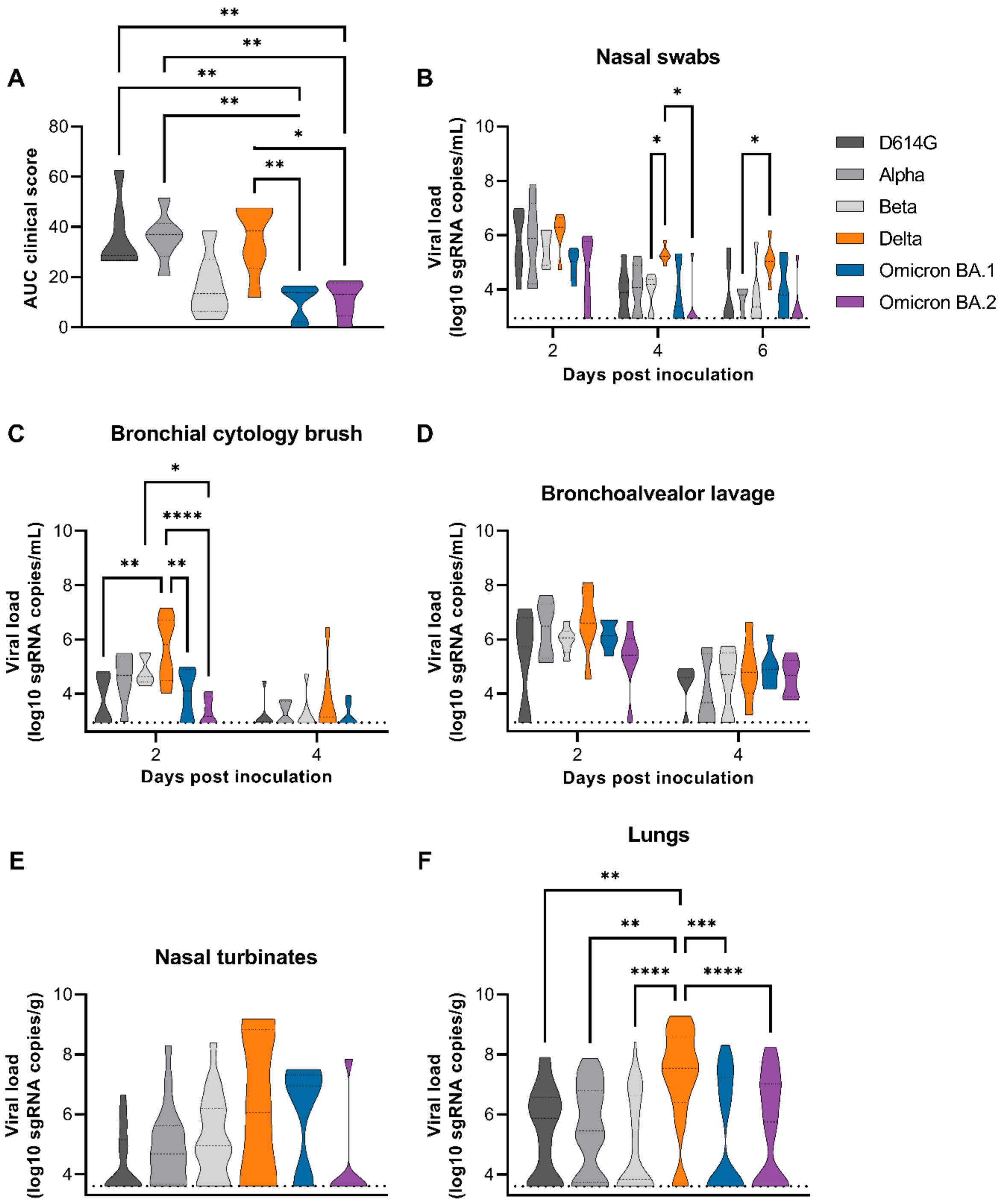

Figure S5. Clinical score and viral load comparison in respiratory tract samples between D614G, Alpha, Beta, Delta, Omicron BA.1, and Omicron BA.2 variants. Truncated violin plot of clinical score (A), viral load in nasal swabs (B), BCBs (C), BAL fluid (D), nasal turbinate tissue (E), and lung tissue (F). Samples in grey are from a previously published study (7). Dotted line = qualitative limit of detection (10 copies per reaction). Statistical analyses done via ordinary one-way ANOVA followed by Holm- Šídák's multiple comparisons test (A), two-way

ANOVA followed by multiple comparison via Tukey (B, C, D), Kruskal-Wallis test followed by multiple comparison via Dunn's (D, E).

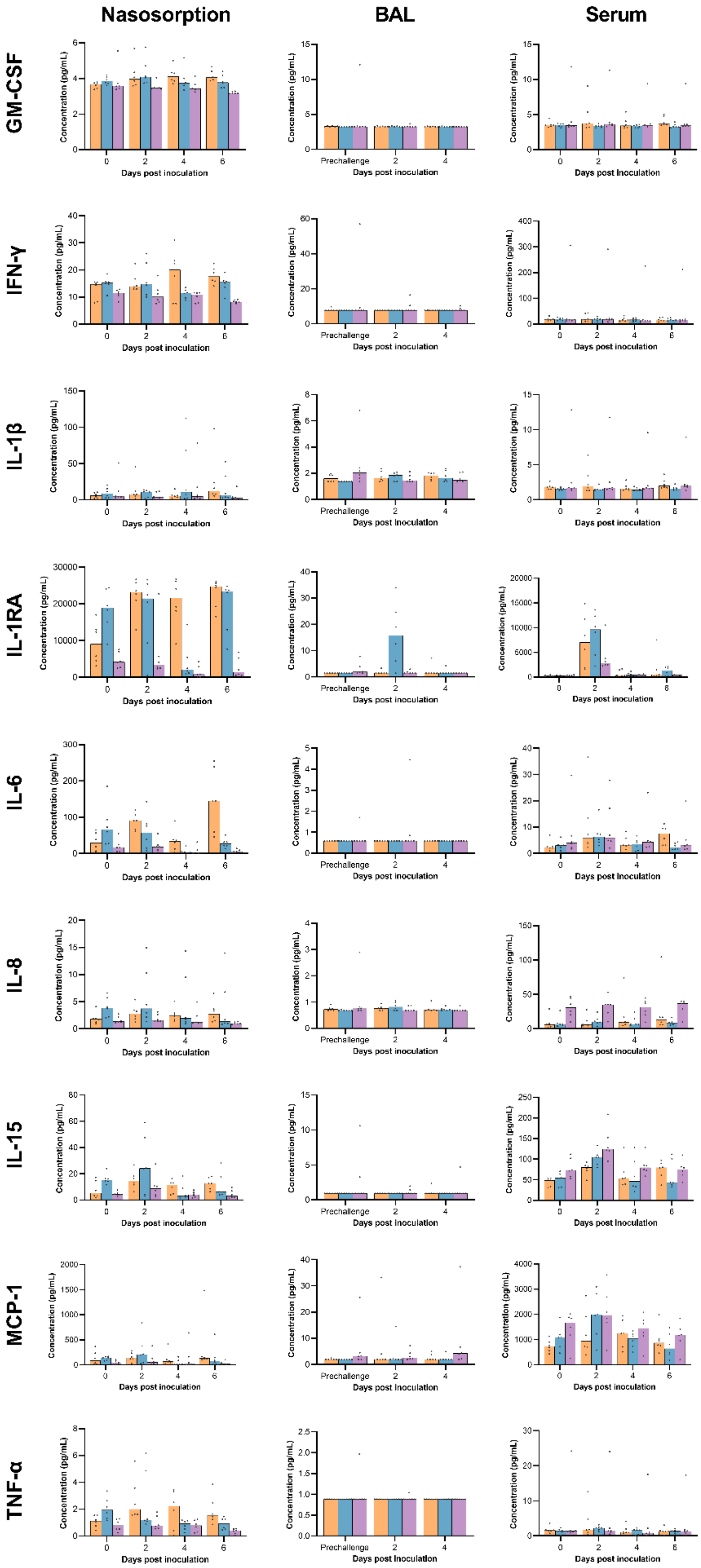

Nasosorption
BAL
Serum
GM-CSF
IFN-γ
IL-1β
IL-1RA
IL-6
IL-8
IL-15
MCP-1
TNF-α
Concentration (pg/mL)
Days post inoculation
Prechallenge

Figure S6. Absolute values of cytokines and chemokines measured in nasosorption, BAL, and serum samples. Bar graphs of median and individual values. Orange = Delta; Blue = Omicron BA.1; Purple = Omicron BA.2
